# Supplementary material for: Enhancing the Biocontrol Potential of the Entomopathogenic Fungus in Multiple Respects via the Overexpression of a Transcription Factor Gene MaSom1
Source: J Fungi (Basel). 2022 Jan 21;8(2):105. doi: 10.3390/jof8020105 (PMC8879238; doi:10.3390/jof8020105)
Supplement: Supplementary file 1 [file jof-08-00105-s001.zip › jof-1442286-supplementary.pdf]

Table S1. Primers used in this study.

Primers	Sequence (5′-3′)	Remarks
MaSom1-F	ATTTCCCCTAAGTACTTCTAGAATGGCCATGAACCCGAATAT	Used to clone the DNA fragment encompassing the *MaSom1* open reading frame
MaSom1-R	TTGCTCACCATGGATCCGTCGGCACCAATCGGGTCGC	
MaSom1-QF	TGGTAATGGCACTGTTAA	Used for detecting the expression level of *MaSom1* gene
MaSom1-QR	TCTGAGGATTATTCTGGTTAT	
Gapdh-QF	AGATGGAGGAGTTGGTGTTG	Used for detecting the expression level of internal control gene
Gapdh-QR	GACTGCCCGCATTGAGAAG	
ITS-F	TGGCATCTTCTGAGTGGTG	Used for measuring the concentration of fungal genomic DNA of hyphal bodies
ITS-R	CCCGTTGCGAGTGAGTTA	
MaSom1-RF	GCTGGCCGCCCATGGGATACAATGGCAACAGGGTCCTA	Used for screening the *MaSom1*-overexpression transformants
EGFP-VR	CCATCACCAGAATCCAACAC	
PgpdA-VF	TCTCCGAAGTAGGTAGAGC	Used for screening the empty transformant
sur-PF	CCTGAAATGTTGTGCTGTC	Used for Southern blot probe1 amplification
sur-PR	CGAGATTATGCCCAAGAAC	

MaUve1-QF	TAACAACGCACGAGATAT	Used for detecting the expression level of *MaUve1* gene
MaUve1-QR	AGGCTATAACCATACTCTG	
MaUbi1-QF	CAAGAAGAAGGTCTACACTACTC	Used for detecting the expression level of *MaUbi1* gene
MaUbi1-QR	AATGTTGCCGTCCTTGTC	
MaSsb1-QF	CTCTCGGTGTCGCTATGG	Used for detecting the expression level of *MaSsb1* gene
MaSsb1-QR	TCGGCAACAGTGGTGAAG	
MaNTH1-QF	TGTTGGCTGGCACTGAGAA	Used for detecting the expression level of *MaNTH1* gene
MaNTH1-QR	ACCAGTCCAAGCAAGCATCT	
MaMpl1-QF	GCTGTCTCTACCGCCTTTGT	Used for detecting the expression level of *MaMpl1* gene
MaMpl1-QR	CTACTGCTTGACCTTCTCCTTGAC	
MaMad2-QF	GCACTATGTCCATCCTTG	Used for detecting the expression level of *MaMad2* gene
MaMad2-QR	TGATGAGGGTCTTGATTTG	
